# A Synopsis of the Symptoms, Diagnosis and Treatment of the More Common and Important Diseases of the Skin

**Published:** 1845-06

**Authors:** 


*A Synopsis of the Symptoms, Diagnosis and Treatment of the more common and important Diseases of the Skin. With sixty coloured Figures.* By N. WORCESTER, M. D., Professor of Physical Diagnosis and General Pathology in the Medical School of Cleveland, late Professor in the Medical College of Ohio. Royal, 8vo. pp. 290. Thomas, Cowperthwait & Co., Philadelphia. Desilver & Brown, Cincinnati, 1845.

We hail with great pleasure the appearance of an *American* work on that most interesting and important, but hitherto too much neglected branch of medicine, *Diseases of the Skin.* It is only a matter of surprise, that this subject should so long have been allowed to remain in a state comparatively unnoticed and uncared for. This is the more remarkable when we consider how much *medical ingenuity* has been called forth, in these latter days, in the getting up of works for the press, either "translated," or "republished with notes and additions," or even "republished" without notes at all! We say it is remarkable, that so fair a field for research and inquiry should so long have been suffered to run to waste by our own *enterprising* countrymen, when there was so reasonable, nay certain prospects of success attending its cultivation.

Although the well read American physician may be familiar with the leading European Authors upon this subject, and may, in some few instances, have made it a topic of special study, still it is perfectly undeniable, that, by the *mass* of our profession, it is one by no means well understood; and one, too, which affords them but little satisfaction when forced upon their attention in the course of their practice. The language of our author is unfortunately but too correct, when he observes of this class of diseases:—"They can scarcely be said to form a part of the professional education of students in the annual courses of medical lectures in this country. It is believed that in many of our medical institutions not a single lecture is given upon the nature, classification and treatment of this interesting class of diseases." "When we add that most of these diseases, when neglected or improperly treated, have a tendency to become unmanageable and rebellious under any subsequent mode of treatment; that morbid changes take place in such cases in the skin and subcutaneous tissues, which last for life, and by the unappeasable irritation the remainder of life is often rendered a burden,—the indifference of our profession is extraordinary. The result has been that these numerous diseases have become an opprobrium to the profession, and a fruitful source of emolument to designing empiricism."

The work before us lays no claim to originality, except perhaps in the author's method of *classification* of cutaneous diseases. Indeed, he frankly informs us in his Preface that "utility and not originality has been his design;" and that (we think very properly) "he has drawn information from every source within his reach."

His mode of discussing the various parts of his subject evidently proves him to have been an attentive student of Willan, Bateman, Alibert, Cazenave, and the other most approved authors on this class of diseases.

The most difficult point to settle with regard to diseases of the skin has ever been their proper method of classification. This has always afforded a fruitful theme for controversy and discussion; and the idea of ever arriving at a *perfect* system—one liable to no objection—cannot, we think, be rationally entertained.

The classification of Willan, the basis of which is the peculiar form which an eruption assumes when at its full maturity, is, we believe, the one most generally adopted both by American and European dermatologists; and there can be no doubt, that for general practical purposes, it is the system most readily learned and most easy of application—particularly for those not skilled in the anatomy and physiology of the skin and its appendages. We, however, fully coincide with Dr. Worcester in his objections to this popular system of classification, on the ground of its want of strict scientific arangement, grouping together, as it does, diseases entirely differing in their nature, pathology, seat, &c., but agreeing in the single point, of resemblance at some one period of the disorder.

Were the present the proper occasion, it would, we think, be easy to substantiate our objections to the Willanian method of classification. One or two examples, however, will be all that we shall adduce in support of them. Take for instance the order *Pustulæ:* this order must necessarily include all those eruptive diseases which, at their period of maturity, present the pustular form. Hence we find *Variola*, a constitutional disease dependent upon a specific contagious principle, classed along with *Impetigo*, a local non-contagious disorder, and *Favus*, a disease peculiar to the hair bulbs; so, also, we find the order *Vesiculæ*, including diseases quite as widely differing in their character—for example, *Scabies, Eczema, and Varicella.* Another objection to Willan's arrangement, arising indeed from the former one, is, that it separates diseases, which are perfectly analogous in their nature, origin, progress, &c. but differing only in the single, comparatively insignificant point,—a want of resemblance at their period of maturity.

Dr. Worsester's method of classification is essentially the same as that of Dr. Willan; although he has so modified it as to embrace all cutaneous affections within two general groups—the "*Dry*," and the "*Moist.*"

The group of "*Moist*" diseases includes three of the orders of Willan, viz. Vesiculæ, Bullæ, and Pustulæ. The group of "*Dry*" diseases includes the remaining five; viz. Exanthemata, Papulæ, Squamæ, Tubercula, and Maculæ. This arangement does not comprehend the *Eruptive Fevers*,—the author considering them as more properly falling under treatises on the practice of medicine. Now, it strikes us that the author's division of cutaneous affections into the two groups of Dry and Moist is a most judicious one for those who prefer the classification of Willan; and by its simplicity it commends itself to us as a decided improvement; still, of course, it must be liable to the same objections we have just urged to the arrangement of Dr. Willan, viz. *a want of strict scientific order.*

Which system of classification then *is* the best? it may be asked. We reply that the one to which we give the decided preference is that advanced by Mr. Erasmus Wilson of London, whose treatise upon this subject we consider one of the most complete ever written.

Wilson's system is based entirely upon the anatomy and physiology of the skin and its appendages; and considers all cutaneous affections solely with reference to these points. Now, these are fixed and unvarying points; they cannot be affected by the different phases through which this or that particular eruption must pass, in its progress; but they afford us a solid groundwork upon which we may build a beautiful system of classification, with certainty and success. Besides, it is strictly a *scientific* arrangement; and the objection which has been urged against it by some–our author among the number—that it is difficult of application in practice, to those not acquainted with the anatomical structure of the dermoid tissue, pre-supposes—what we conceive should never exist—an ignorance on this subject on the part of the practitioner. The objection would equally well apply to every sort of classification—for instance the classification of medicines in the Materia Medica. Who could properly understand the modus operandi of a purgative, an emetic or an expectorant, without a previous knowledge of the anatomy and physiology of the bowels, the stomach and the lungs?

But our remarks have extended much further than we had designed. We will only add, that the work before us is beautifully printed, and is creditable both to its author and to the Cincinnati press whence it has issued. We should have been glad for our own credit, if *Philadelphia*, and not a city beyond the mountains, had given it birth; although, at the same time, we congratulate our western brethren upon its appearance. As regards the *plates*, we are sorry we cannot say much in their favor: they are mostly copies from Rayer—but they are perfect crudities when compared with their Parisian original, and are not at all in keeping with the practical excellence of the other parts of the book.

